# The cannabinoid receptor 1 is involved in renal fibrosis during chronic allograft dysfunction: Proof of concept

**DOI:** 10.1111/jcmm.14570

**Published:** 2019-08-30

**Authors:** Myriam Dao, Lola Lecru, Sophie Vandermeersch, Mélanie Ferreira, Sophie Ferlicot, Katia Posseme, Antoine Dürrbach, Bogdan Hermeziu, Charlotte Mussini, Christos Chatziantoniou, Hélène François

**Affiliations:** ^1^ Inserm UMR_S 1155 Hôpital Tenon Paris France; ^2^ APHP, Service de Néphrologie Adulte Hôpital Necker Paris France; ^3^ Galapagos SASU Romainville France; ^4^ AP‐HP, Service d'Anatomie et de Cytologie Pathologiques Hôpital Bicêtre, Université Paris Sud Le Kremlin Bicêtre France; ^5^ AP‐HP, Service de Néphrologie Hôpital Bicêtre, Université Paris Sud Le Kremlin Bicêtre France; ^6^ AP‐HP, Service d'Hépatologie Pédiatrique Hôpital Bicêtre Le Kremlin Bicêtre France; ^7^ AP‐HP, Unité de Néphrologie et de Transplantation rénale, Hôpital Tenon Sorbonne Université Paris France

**Keywords:** cannabinoid receptor 1, chronic allograft dysfunction, renal fibrosis, renal transplantation

## Abstract

Chronic allograft dysfunction (CAD), defined as the replacement of functional renal tissue by extracellular matrix proteins, remains the first cause of graft loss. The aim of our study was to explore the potential role of the cannabinoid receptor 1 (CB1) during CAD. We retrospectively quantified CB1 expression and correlated it with renal fibrosis in 26 kidney‐transplanted patients who underwent serial routine kidney biopsies. Whereas CB1 expression was low in normal kidney grafts, it was highly expressed during CAD, especially in tubular cells. CB1 expression significantly increased early on after transplantation, from day 0 (D0) to month 3 post‐transplant (M3) (22.5% ± 15.4% vs 33.4% ± 13.8%, *P* < .01), and it remained stable thereafter. CB1 expression correlated with renal fibrosis at M3 (*P* = .04). In an in vitro model of tacrolimus‐mediated fibrogenesis by tubular cells, we found that tacrolimus treatment significantly induced mRNA and protein expression of CB1 concomitantly to *col3a1* and *col4a3* up regulation. Administration of rimonabant, a CB1 antagonist, blunted collagen synthesis by tubular cells (*P* < .05). Overall, our study strongly suggests an involvement of the cannabinoid system in the progression of fibrosis during CAD and indicates the therapeutic potential of CB1 antagonists in this pathology.

## 1 INTRODUCTION

The progressive and inevitable impairment of renal graft function, called chronic allograft dysfunction (CAD), remains the first cause of graft loss.^1^ CAD corresponds to the replacement of functional renal tissue by extracellular matrix (ECM) proteins, mainly collagens, leading to both interstitial fibrosis and tubular atrophy (IF/TA). Other histological damages include glomerulosclerosis, splitting of glomerular capillary basement membranes and vascular intimal hyperplasia.^2^ Because this process is multifactorial and complex, there is still to date no efficient treatment of CAD.^3^, ^4^, ^5^, ^6^, ^7^ CAD is a multifactorial process in which a lot of immunological and non‐immunological causes are involved,^3^, ^4^, ^5^, ^6^, ^7^ related to donor,^8^, ^9^ recipient, organ conservation and transfer,^10^ surgery, rejection, recurrence of primary renal diseases… Antibody‐mediated rejection has recently emerged as one of the major cause of CAD.^7^, ^11^, ^12^ Calcineurin inhibitor (CNI) nephrotoxicity has been considered for a long time as the most prominent cause of renal allograft failure, but its role may have been overstated. However, CNI toxicity is well demonstrated in other organ recipients and in native kidneys.^13^, ^14^, ^15^, ^16^ In the specific case of renal transplantation, CNI nephrotoxicity can produce renal fibrosis but progression to graft failure from CNI nephrotoxicity alone is uncommon.^7^, ^11^ However, renal fibrogenesis leading to IF/TA during CAD resembles what is seen during chronic kidney disease (CKD) in native kidneys. Therefore, a better understanding of renal fibrogenesis during CAD is an essential therapeutic approach.^17^

We and others have recently showed the potential role of the endocannabinoid system, and especially of the cannabinoid receptors 1 and 2 (CB1, CB2) in renal metabolic and non‐metabolic disease.^18^, ^19^, ^20^, ^21^, ^22^, ^23^, ^24^ CB1 is best known to be involved in the regulation of behaviour in the central nervous system and in metabolic pathways in peripheral tissues 25, 26, 27 whereas CB2 is mainly expressed in the immune system.^28^ Whereas expression of CB1 is low in normal kidneys,^18^, ^29^, ^30^ we previously found that its expression is increased in IgA nephropathy, acute interstitial nephritis and diabetic nephropathy.^18^ Experimental studies in animals showed that in injured kidneys, CB1 is expressed in various structures in glomeruli,^18^, ^19^, ^20^, ^21^, ^31^ especially in podocytes and mesangial cells, in the tubules,^18^, ^32^ in the interstitium^18^ and in vessels.^18^ Recent studies found that CB1 is involved in the development of renal disease during diabetes and/or obesity,^19^, ^21^, ^22^, ^23^, ^24^ both by its role on metabolism and through a direct action in podocytes and tubules. Our group previously demonstrated for the first time an anti‐fibrotic role of CB1 blockade in non‐metabolic experimental renal fibrosis in mice in the unilateral ureteral obstruction (UUO) model.^18^ In this model, both the pharmacological blockade and the genetic disruption of CB1 profoundly reduced the development of renal fibrosis. This effect was mainly due to a direct paracrine/autocrine role of CB1 in myofibroblasts, which are the final effector cells in renal fibrogenesis. We found that upon TGFβ stimulation, renal myofibroblasts expressed CB1 and secreted endocannabinoid ligands, whereas CB1 blockade reduced collagen synthesis. These results suggest that the CB1 pathway may be a major target against the development of renal fibrosis in various types of renal injury.^33^

The aim of the present study was to examine whether the activation of the CB1 receptor is involved in the progression of renal fibrosis during CAD. In the first part of our work, we quantified CB1 expression by immunohistochemistry (IHC) and a morphometry software, and we correlated it with renal fibrosis, Banff scoring and clinical data. In the second part, we studied the in vitro expression of CB1 after tubular injury induced by tacrolimus and the effects of rimonabant, a CB1 antagonist, in tacrolimus‐induced fibrogenesis, which we used as an in vitro model of CAD.

## 2 MATERIALS AND METHODS

### 2.1 Patients and kidney graft biopsies

We retrospectively included all kidney‐transplanted patients in Bicêtre hospital who received a kidney graft in 2012 and 2013 and who underwent a routine kidney graft biopsy at day 0 (D0), month 3 (M3) and month 12 (M12). Kidney donation followed the 2008 Declaration of Istanbul principles and the French Agence Nationale de la Biomedecine regulation. Informed written consent was given by the patients for the use of part of the biopsy for scientific purposes. All procedures and the use of tissues were performed in accordance with the Declaration of Helsinki principles. We reviewed clinical reports from the 26 patients. Biological tests were performed on blood samples harvested concurrently with the kidney allograft biopsy. Kidney graft biopsies were processed for routine light microscopy. Biopsy samples were fixed in formalin, acetic acid and alcohol (FAA) and sliced 3 µm thick. The slides were stained with Masson's trichrome, haematoxylin, eosin and saffron (HES), periodic acid Schiff and Jones methenamine silver stains. Biopsies were reviewed for histological features according to 2018 Banff recommendations.^2^ Semi‐quantitative scoring for acute and chronic lesions (glomerulitis, peritubular capillaritis, interstitial inflammation, total inflammation, tubulitis, intimal arteritis, allograft glomerulopathy, mesangial matrix increase, interstitial fibrosis, tubular atrophy, vascular fibrous intimal thickening and arteriolar hyaline thickening) and C4d immunostaining provided the morphologic basis for main diagnosis classification: normal biopsy or nonspecific changes (after exclusion of any diagnosis from the Banff Diagnostic Categories), antibody‐mediated changes, suspicious (borderline) for acute T cell–mediated rejection, T cell–mediated rejection, IF/TA, other changes not considered to be caused by acute or chronic rejection (such as BK‐virus nephropathy, CNI toxicity, acute tubular injury, recurrent disease, de novo glomerulopathy other than transplant glomerulopathy…).^2^

### 2.2 Histopathological analysis of renal fibrosis

Interstitial fibrosis (ci) and tubular atrophy (ct) were separately assessed using the Banff classification: respectively ci0 = interstitial fibrosis in up to 5% of cortical area; ci1 = interstitial fibrosis in 6 to 25% of cortical area (mild interstitial fibrosis); ci2 = interstitial fibrosis in 26 to 50% of cortical area (moderate interstitial fibrosis); ci3 = interstitial fibrosis >50% of cortical area (severe interstitial fibrosis) and ct0 = no tubular atrophy; ct1 = tubular atrophy in up to 25% of the area of cortical tubules; ct2 = tubular atrophy involving 26 to 50% of the area of cortical tubules; ct3 = tubular atrophy in >50% of the area of cortical tubules. Taking together, ci and ct allowed to semi‐quantitatively grade IF/TA according to Banff classification: grade I, mild (ci1 or ct1); grade II, moderate (ci2 or ct2); and grade III, severe (ci3 or ct3).^2^ To improve accuracy of interstitial fibrosis evaluation, renal biopsy sections (3 µm) were stained with Sirius red. Sirius red specifically stains collagen fibres. Renal cortex fibrosis was quantified using a computer‐based morphogenic analysis software (Calopix, Tribvn, Montrouge, France). The red positive area was expressed as a percentage of the entire cortical kidney section.

### 2.3 Cannabinoid receptor 1 immunohistochemical staining

Cannabinoid receptor 1 immunohistochemical staining was performed using rabbit polyclonal anti‐CB1 antibody (Abcam; dilution 1/100). Epitope retrieval was achieved using heat‐mediated retrieval method in citrate buffer (10 mmol/L Sodium Citrate, 0.05% Tween 20, pH 6.0). Endogenous peroxidase activity was blocked by 0.3% hydrogen peroxide in methanol for 10 minutes. Primary CB1 antibody was incubated for 2h at room temperature. Horseradish peroxidase (HRP) secondary antibody was applied for 1h at room temperature. Staining was revealed by applying a 3'‐diaminobenzidine (DAB) kit (DakoCytomation). Appropriate positive and negative controls were run concurrently. Morphometric analyses and quantification were quantified using computer‐based morphogenic analysis software (Calopix, Tribvn,). The brown positive area was expressed as a percentage of the entire cortical kidney section.

### 2.4 Cell cultures and treatments

Human proximal tubule epithelial cells (HK‐2) from American Type Culture Collection (ATCC) were cultured in Quantum 286 (Medium for epithelial cells, PAA, France) supplemented with 1% (v/v) penicillin/streptomycin, at 37°C in 95% air–5% CO2. The cells were subcultured at 80% confluence using 0.05% trypsin with 0.02% EDTA. Cells were used between passages 15 to 17. Murine proximal tubular epithelial cells (mPTEC) were isolated and cultured from C57/BL6 mice, as previously described.^34^ Cells were used between passages 5 to 9.

Tacrolimus was preserved as a stock solution (PROGRAF 5 mg/mL, Astellas). Working solution was prepared by extemporaneous mixture of stock solution in 100% ethanol. Rimonabant (Sanofi‐Aventis R&D) was preserved as a stock solution (10 mmol/L). Working solution was prepared by extemporaneous mixture of stock solution in DMSO.

### 2.5 Real‐time quantitative PCR (RT‐qPCR)

RNA was extracted from cells cultures using EZ‐10 Spin Column Total RNA Mini‐preps Super Kit (Bio Basic Inc) according to the manufacturer's instructions. RNA concentration was measured by NanoDrop1000 spectrophotometer (Thermo Scientific). cDNA was synthesized using Maxima First Strand cDNA Synthesis Kit (Thermo scientific), and PCR was performed using SYBR green and specific primers (Table 1) on a Light Cycler 480 (Roche). Expression levels were normalized to the house‐keeping gene GAPDH using Lightcycler advanced relative quantification programme (Roche).

**Table 1:** Primers (Eurogentec) used for RT‐qPCR

mRNA	Strand	Sequence
cnr1	Sense	5′‐GGGCAAATTTCCTTGTAGCA‐3′
	Antisense	5′‐GGCTCAACGTGACTGAGAAA‐3′
col3a1	Sense	5′‐TCCCCTGGAATCTGTGAATC‐3′
	Antisense	5′‐TGAGTCGAATTGGGGAGAAT‐3′
col4a3	Sense	5′‐CACGGGTTCCAAAGGTGTAA‐3′
	Antisense	5′‐AGTCCGTAAGGCCCGGTAT‐3′
gapdh	Sense	5′‐AGCTTGTCATCAACGGGAAG‐3′
	Antisense	5′‐TTTGATGTTAGTGGGGTCTCG‐3′

### 2.6 Western blotting

Cells were lysed in PhosphoP38 buffer containing protease inhibitors (Roche, Mannheim, Germany) and quantified by Bradford's method. Proteins were separated in a 7% SDS‐PAGE gel and transferred to a polyvinylidene fluoride membrane. Immunoblotting was performed using a rabbit anti CB1 (Abcam) diluted 1:800 or a rabbit beta‐actin diluted 1:5000 for loading control. The membranes were then probed with a HRP‐conjugated secondary antibody diluted 1:5,000 (Amersham), and the bands were detected by enhanced chemiluminescence using ECL Plus (Amersham). A PXi (Syngen) imaging system was used to reveal bands, and densitometric analysis was used for quantification. Membranes were then incubated with anti‐rabbit antibody conjugated to HRP (Millipore), and proteins were visualized by the addition of chemiluminescent HRP substrate (Immobilon Western, Millipore).

### 2.7 Sirius red collagen assay

Total collagen content in the culture cell supernatant was quantified using the Sirius red collagen detection kit (Chondrex) according to the manufacturer's instructions. Each supernatant was diluted in 1:2.5 in 0.05 mol/L. Optical density was read at 530 nm against the reagent blank using a spectrophotometer (Xenius, SAFAS, Monaco).

### 2.8 Statistical analyses

Descriptive statistical methods (means, medians, standard deviations and ranges) were used to assess the distributions of variables. Associations between categorical variables were assessed with Fisher's exact test (2 groups) or chi‐square test (3 groups). Associations between quantitative variables were assessed with Mann‐Whitney test (2 groups) or Kruskal‐Wallis test (3 groups). Correlations between quantitative variables were assessed with Pearson product‐moment correlation coefficient. For all analyses, a *P* value <.05 was regarded as significant. Analyses were performed using the R software (version 3.2.0) and GraphPad 5.0.^35^

## 3 RESULTS

### 3.1 Patients

We selected patients transplanted in Bicêtre hospital between 2012 and 2013 who underwent a routine kidney biopsy at D0, M3 and M12. We included 26 patients in our study. The patients included 11 females and 15 males. The mean age at the time of kidney transplantation was 54 ± 13 years. The indications for kidney transplantation were hypertensive nephrosclerosis and/or diabetic nephropathy (n = 8), other glomerulopathies (n = 4), tubulointerstitial nephritis (n = 3), uropathy (n = 3) and autosomal dominant polycystic kidney disease (n = 2). Nephropathy remained undetermined in 3 patients. Patients received induction therapy with anti‐lymphocyte serum or basiliximab. They also received mycophenolate mofetil, corticosteroids and tacrolimus per local practice (mean through tacrolimus level at M3: 9.0 ± 3.9 ng/mL and at M12: 7.8 ± 4.4 ng/mL). Four patients received belatacept in place of calcineurin inhibitors. All patients received a kidney graft from a deceased donor. Among the donors, 22 were brain‐dead donors (8 standard donors [SD] and 14 extended criteria donors [ECD]) and 4 were cardiac‐dead donors (CDD) deceased after unforeseeable irreversible circulatory arrest (Maastricht 2). Donor age, history of diabetes or active smoking, use of catecholamines and serum creatinine were similar among the different groups of donors. As expected, vascular causes of deaths and prevalence of high blood pressure were more frequent in brain‐dead donors (respectively, SD 75%, ECD 71% vs CDD 0%, *P* = .02 and SD 63%, ECD 86% vs CDD 0%, *P* < .01).

### 3.2 Histological features of renal biopsies at D0, M3 and M12 and renal graft function

Histological features of the 78 renal biopsies, reviewed according to the 2018 Banff recommendations, are summarized in Table 2. They were similar by donor type (Table S1). In preimplantation biopsies, IF/TA was absent or mild in 24/26 samples (92%), respectively 10/26 (38%), and 14/26 (54%) biopsies. Preimplantation biopsies also exhibited mild to moderate lesions of arteriosclerosis (cv1 or cv2, 62%) and acute tubular necrosis (85%). The most relevant findings were a significant decrease of acute tubular necrosis (ATN) over time (85% at D0% vs 19% at M3 and M12, *P* < .01) and a significant increase of renal graft fibrosis (0.6 ± 0.7 at D0 vs 1.1 ± 0.9 at M3 and 1.3 ± 1.0 at M12, *P* = .04). At M3, mean creatininemia was 143 ± 59 µmol/L (median: 121 µmol/L, ranges: 69‐289 µmol/L) and mean estimated glomerular filtration rate (eGFR) was 47 ± 19 mL/min/1.73 m^2^ by CKD‐EPI. At M12, mean creatininemia slightly increased at 160 ± 75 µmol/L (median: 127 µmol/L, ranges: 69‐389 µmol/L) and mean GFR slightly decreased at 41 ± 19 mL/min/1.73 m^2^ (*P* = .5). Taken together, both the significant increase in IF/TA and the decline in eGFR established that CAD was developed in these patients.

**Table 2:** Histological features of renal biopsies at D0, M3 and M12

	D0 (n = 26)	M3 (n = 26)	M12 (n = 26)	*P*
Specimen adequacy according to the Banff recommendations				
Adequate samples (n)	19	15	9	.019
Limit samples (n)	6	7	11	.28
Inadequate samples (n)	1	4	6	.13
Acute lesions				
Glomerulitis «g»	NA	0.31 ± 0.55	0 ± 0	<.01
Peritubular capillaritis «ptc»	NA	0.15 ± 0.37	0.083 ± 0.41	.23
Interstitial inflammation «i»	NA	0.042 ± 0.20	0.087 ± 0.29	.55
Total inflammation «ti»	NA	0.38 ± 0.74	0.5 ± 0.72	.41
Tubulitis «t»	NA	0.08 ± 0.28	0.4 ± 0.71	.059
Intimal arteritis «v»	NA	0 ± 0	0 ± 0	NA
Acute tubular necrosis	85% (n = 22)	19% (n = 5)	19% (n = 5)	<.01
Chronic lesions				
Sclerotic glomeruli	10.3% ± 11.3%	9.1% ± 11.6%	13.9% ± 13.7%	.42
Allograft glomerulopathy «cg»	0 ± 0	0.08 ± 0.28	0.042 ± 0.20	.15
Mesangial matrix increase «mm»	0.12 ± 0.59	0.42 ± 0.86	0.42 ± 0.88	.077
Interstitial fibrosis «ci»	0.64 ± 0.7	1.08 ± 0.93	1.33 ± 0.96	.040
Tubular atrophy «ct»	0.4 ± 0.71	0.81 ± 0.75	1.13 ± 0.95	.083
Vascular fibrous intimal thickening «cv»	0.92 ± 0.89	0.76 ± 0.78	1.32 ± 0.99	.62
Arteriolar hyaline thickening «ah»	0.56 ± 0.92	0.69 ± 0.84	0.8 ± 0.91	.33
C4d (immunofluorescence)				
Negative (0 or minimal 1)	NA	88% (22/25)	92% (22/24)	1
Positive (focal 2 or diffuse 3)	NA	12% (3/25)	8% (2/24)	1
Rejection				
Antibody‐mediated rejection	NA	12% (n = 3)^a^	0	.24
Borderline rejection	NA	8% (n = 2)	23% (n = 6)	.13
T cell–mediated rejection	NA	0	0	NA
Other pathologies	0	2^b^	8^c^	

Note

Digital data are means ± standard deviation.

a Antibody‐mediated rejection included 1 acute antibody‐mediated rejection, 1 chronic active antibody‐mediated rejection and 1 ‘suspicious’ for acute antibody‐mediated rejection.

b Other pathologies were 1 diabetic glomerulonephritis and 1 recurrence of focal segmental glomerulosclerosis.

c Other pathologies were 1 diabetic glomerulonephritis, 2 BK‐polyomavirus‐associated nephropathy, 1 de novo membranous glomerulonephritis, 2 focal segmental glomerulosclerosis (including 1 recurrence of original disease), 1 acute bacterial pyelonephritis and 1 thrombotic micro‐angiopathy.

### 3.3 Cannabinoid receptor 1 expression in kidney transplants during chronic allograft dysfunction

**Figure 1:**
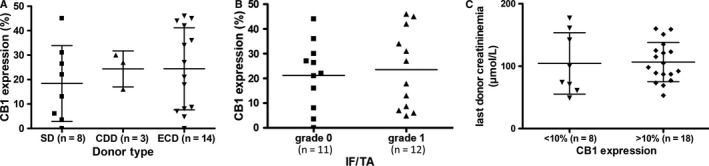
Cannabinoid receptor 1 expression on preimplantation biopsies was not associated with donor characteristics. A, CB1 expression was similar by donor type. B, CB1 expression was not associated by IF/TA on preimplantation biopsies. C, Donor creatininemia at time of organ removal was not associated with CB1 expression. Abbreviations: CDD, cardiac death donor; ECD, extended criteria donor; IF/TA, interstitial fibrosis and tubular atrophy; SD, standard donor

**Figure 2:**
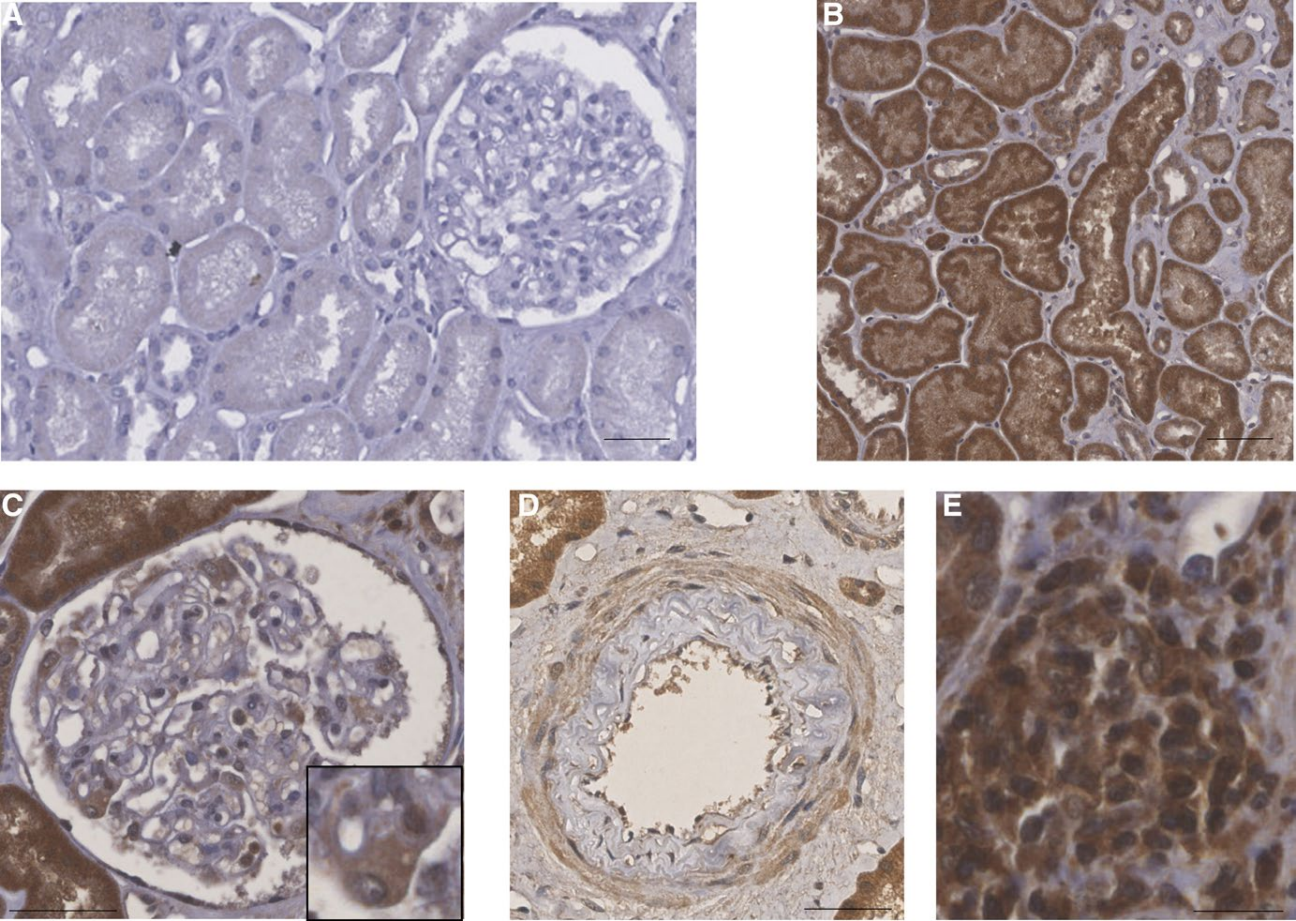
Cannabinoid receptor 1 expression in 3 mo biopsies of renal grafts developing CAD revealed by peroxidase immunohistochemistry. A, Absence of CAD: CB1 was not expressed. The biopsy was performed in a 66‐y‐old man who received a kidney graft from extended criteria donor. Early allograft history was unremarkable. M3 graft biopsy exhibited a normal graft according to the Banff classification, without lesions nor IF/TA. M3 creatininemia was 128 µmol/L and immunosuppressive regimen included prednisone, mycophenolate mofetil and tacrolimus (T0 4.5 ng/mL). B, CB1 expression in tubular epithelial cells. C, CB1 glomerular expression is mainly found in the podocyte (in the inset). D, CB1 expression in medium‐sized arteries: endothelial cells, smooth muscle cells of media and to a lesser extent intimal cells express CB1. E, CB1 expression in the interstitial inflammatory infiltrate. Bar scales = 50 µm. Abbreviations: CAD, chronic allograft dysfunction; IF/TA, interstitial fibrosis and tubular atrophy

**Figure 3:**
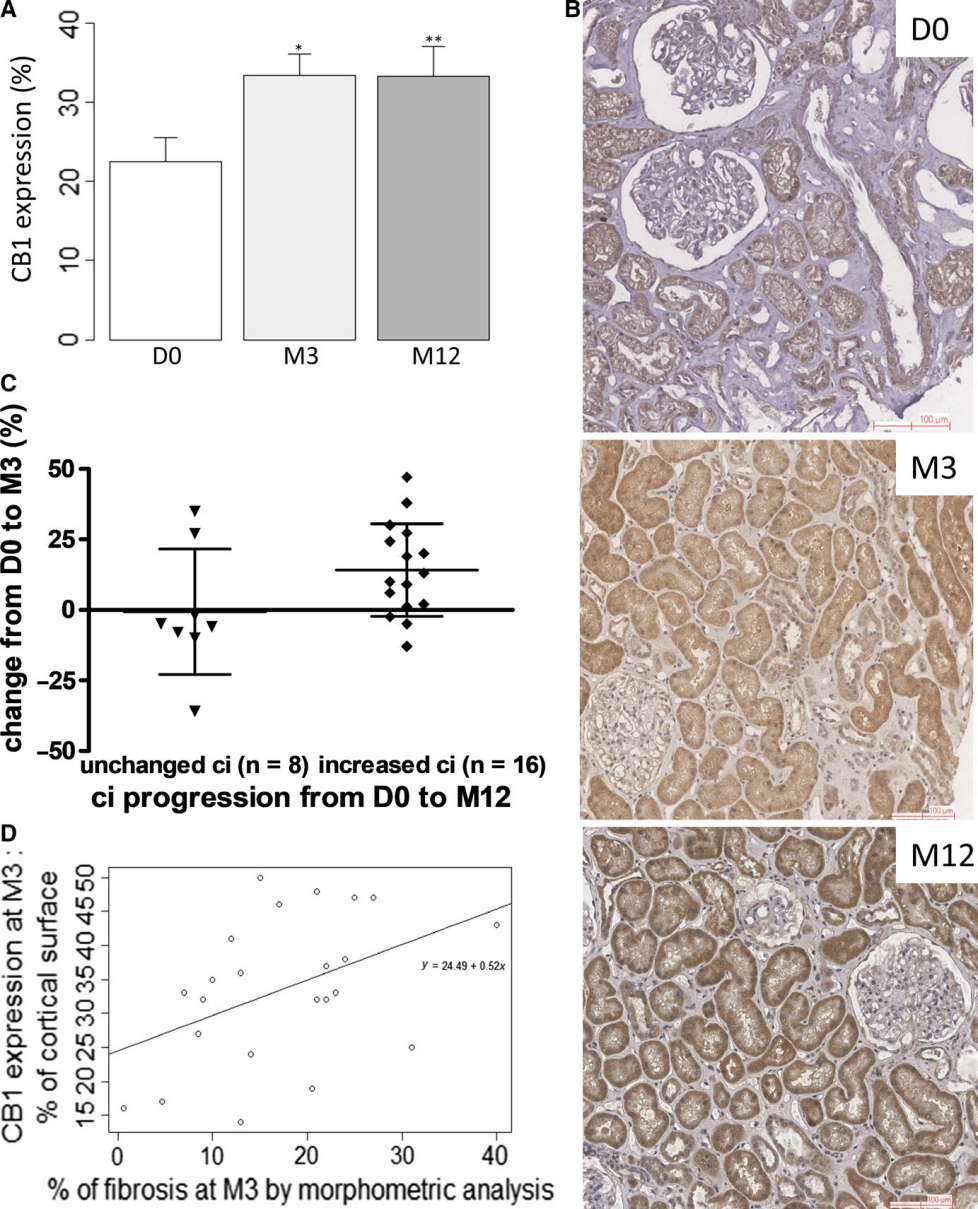
Cannabinoid receptor 1 expression is induced during CAD and correlates with renal fibrosis. A, Quantification of CB1 expression by a morphometry software (Calopix, Tribvn). *P < .05 and **P < .05 compared to D0. Means ± standard deviation. B, Illustrative case of CB1 expression at D0, M3 and M12 revealed by peroxidase immunohistochemistry. The biopsies were performed in a 68‐y‐old woman who received a kidney graft from an extended criteria donor. Preimplantation biopsy was normal according to Banff classification, with IF/TA grade 0. Creatininemia at organ removal was 89 µmol/L. From M3, graft biopsies exhibited IF/TA grade 1 according to the Banff classification, without other changes. At M3 and M12, creatininemia were, respectively, at 155 µmol/L and 122 µmol/L. Her immunosuppressive regimen included prednisone, mycophenolate mofetil and tacrolimus (T0 3.2 ng/mL at M3 and 4.3 ng/mL at M12). Quantification of CB1 expression by a morphometry software was 21% at D0, 48% at M3 and 57% at M12. C, Patients with unchanged ci from D0 to M12 tended to have less CB1 progression evaluated by a morphometry software (Calopix, Tribvn) (−0.62 ± 7.8 vs 14.1% ± 4.1%, *P* = .08). D, Correlation between CB1 expression and renal fibrosis assessed by morphometry software (Calopix, Tribvn) at M3 (n = 26, NA = 3, Pearson correlation test, *P* = .04, *R* = .44). Abbreviations: CAD, chronic allograft dysfunction; D0, day 0; IF/TA, interstitial fibrosis and tubular atrophy; M3, month 3; M12, month 12

We found that 23% ± 15% of cortical area was positive for CB1 staining at D0 in preimplantation biopsies. CB1 expression at D0 was similar by donor type and by IF/TA grade. It was not associated with donor last creatininemia (Figure 1). At M3 and M12, whereas CB1 expression was low in normal graft, it was induced in many cell types during CAD (Figure 2) such as proximal and distal tubular epithelial cells, medium‐sized arteries and arterioles vascular smooth muscle cells, interstitial inflammatory infiltrate and glomeruli, mainly podocytes. Specifically, CB1 expression significantly increased from D0 to M3 (23% ± 15% of stained cortical area vs 33% ± 14%, *P* = .01) and then it remained increased up to M12 (33% ± 19% of stained cortical area) (Figure 3A and 3). Patients with stable interstitial fibrosis from D0 to M12 tended to have lower CB1 progression (n = 8, CB1 expression −0.62% ± 7.8%) than patients in whom interstitial fibrosis increased (n = 16, 14.1% ± 4.1%, *P* = .08) (Figure 3C). We also found a positive correlation between CB1 expression and renal fibrosis at M3 (*P* = .04, *R* = .44) but not at M12 (Figure 3D). This result may be due to a lack of power (only 35% biopsies were adequate according to Banff classification at M12). However, we did not find a significant correlation between CB1 expression and eGFR.

### 3.4 Cannabinoid receptor 1 expression in the tacrolimus‐induced model of tubule injury in vitro

**Figure 4:**
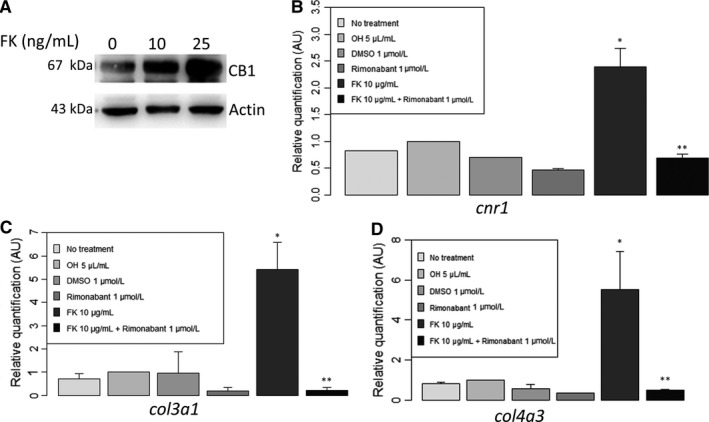
In vitro model of tacrolimus‐mediated fibrogenesis: tacrolimus increased CB1 expression, *cnr1* (encoding for CB1) expression as well as *col3a1* (encoding for Collagen 3) and *col4a3* (encoding for Collagen 4). *Cnr1*, *col3a1* and *col4a3* expression were significantly blunted by rimonabant, a CB1 antagonist. A, Tacrolimus significantly increased CB1 expression (Western blot, n = 4, 3.5 ± 3.4 vs 1.0 ± 0, relative quantification after normalization, *P* = .03) in human proximal tubule epithelial cells (HK2) after 48 h of daily treatment with tacrolimus (FK) (10 ng/mL and 25 ng/mL). B, Expression of *cnr1* mRNA evaluated by RT‐qPCR after 24 h of treatment (n = 4 for each group). *P < .05 vs ethanol 5 µL/mL. **P < 0.05 vs FK 10 µg/mL. C, Expression of *col3a1* mRNA evaluated by RT‐qPCR after 24 h of treatment (n = 4 for each group). *P < 0.05 vs ethanol 5 µL/mL. **P < .05 vs FK 10 µg/mL. D, Expression of *col4a3* mRNA evaluated by RT‐qPCR after 24 h of treatment (n = 4 for each group). *P < .05 vs ethanol 5 µL/mL. **P < .05 vs FK 10 µg/mL

We next studied the role of CNI in CB1 expression using HK‐2 as a model of CAD. We hypothesized that tubular stress induced by CNI would increase CB1 expression. Protein expression was evaluated by Western blot analysis. Tacrolimus significantly increased CB1 expression (n = 4, 3.5 ± 3.4 vs 1.0 ± 0, relative quantification after normalization, *P* = .03) (Figure 4). This result was confirmed by RT‐qPCR analysis on mPTEC. We found a significant increase in *cnr1* (encoding for CB1) expression after 24 hours of treatment with tacrolimus (n = 4, 2.4 ± 0.7 vs 1.0 ± 0, relative quantification after normalization, *P* = .02).

### 3.5 Cannabinoid receptor 1 blockade decreased expression of mesenchymal markers on mPETC in the tacrolimus‐induced model of renal injury

In addition, tacrolimus administration on epithelial tubular cells increased not only CB1 expression but also *col3a1* (encoding for collagen III) and *col4a3* (encoding for collagen IV) (Figure 4) and total collagen in cell supernatants (Figure S1). Addition of rimonabant, a CB1 inverse agonist, strongly blunted *col3a1 and col4a3* expressions (Figure 4) and decreased total collagen in cell supernatants (Figure S1).

## 4 DISCUSSION

The general objective of our research is to find new pathways in the development of renal interstitial fibrosis which is a key feature of CAD. In the present study, we establish for the first time an interaction between abnormal CB1 expression and progression of renal fibrosis, leading to CAD. We and others have previously published that CB1 is a major mediator in both metabolic renal disease 22, 23, 24 and non‐metabolic renal fibrosis,^18^ but its expression was never assessed in renal grafts. In our work, we found that 23% ± 15% of cortical area was positive for CB1 staining at D0 in preimplantation biopsies whereas IF/TA was absent or mild in most of preimplantation biopsies. Out of the 26 graft D0 biopsies, 10/26 (38%) showed no IF/TA and 14/26 (54%) mild IF/TA according to the Banff classification. In our previous study,^18^ we found a low level of CB1 expression (6.5% ± 4.8%, n = 5) in normal kidneys, which is lower than the D0 biopsies (ie 23% ± 15%). However, the preimplantation biopsies of our series do not correspond to the ‘normal’ category of our previous paper since they were performed at the end of the cold preservation period just before graft transplantation and as expected revealed significant ATN, which is the consequence of ischaemia (22/26, 85% revealed ATN). Indeed, previous studies described the metabolic consequences of ischaemia: compromised mitochondrial ATP production and activation of anaerobic glycolysis leading to ATN.^36^, ^37^, ^38^, ^39^ Therefore, the high level of D0 CB1 expression that we observed is not associated with concurrent IF/TA but is a consequence of cold ischaemia‐induced ATN. In addition, recent studies demonstrated that renal hypoxia‐induced ATN promotes tubulointerstitial fibrosis.^40^, ^41^, ^42^ Hence, our hypothesis is that CB1 expression at D0 is predictive for the development of kidney graft fibrosis as a consequence of ischaemia‐induced ATN and that early CB1 expression could be used as a biomarker.

We next studied CB1 expression at M3 and M12. CB1 expression was low in normal kidney grafts, similar to CB1 expression in normal native kidneys. Interestingly, we found that CB1 was induced in many different cell types during CAD: tubular epithelial cells, medium‐sized arteries (endothelial cells, smooth muscle cells of media), interstitial inflammatory infiltrate and glomeruli (mainly in podocytes). During CAD, CB1 expression significantly increased early on after transplantation, from D0 to M3 and it remained stable and high (around 30% of the total cortical area of the kidney graft) thereafter. This high expression corresponds to a plateau of CB1 expression which is reached at M3. It is noteworthy that in CAD, CB1 expression was higher (33% from M3) than in native nephropathies (18%) 18 and found mostly in tubules. We also found a parallel increase of IF/TA from D0 to M3, in accordance with the literature regarding the development of IF/TA assessed by routine kidney biopsies.^43^ We found that not only CB1 and IF/TA increased from D0 to M3 in kidney grafts but also that there was a significant positive correlation between CB1 expression and renal fibrosis at M3 (*P* = .04). Moreover, individual CB1 expression trajectories from D0 to M3 then M12 allowed to distinguish groups of patients: patients with stable interstitial fibrosis from D0 to M12 tended to have lower CB1 progression (n = 8, CB1 expression −0.62% ± 7.8%) than patients in whom interstitial fibrosis increased (n = 16, 14.1% ± 4.1%, *P* = .08). Therefore, CB1 could be a key player in the early steps of the development of IF/TA in kidney grafts or at least be a marker of renal fibrosis. Conversely to what we found in a small cohort of patients with various nephropathies in native kidneys,^18^ we could not establish that CB1 expression correlated with kidney graft function. This difference can be due to the slow decline of kidney graft function in our CAD cohort as well as the short‐term follow‐up. However, the development of IF/TA usually precedes kidney graft dysfunction. As previous studies demonstrated that early interstitial fibrosis was associated with chronic allograft dysfunction and kidney graft outcome,^7^, ^44^, ^45^, ^46^ early increased CB1 expression may be an early event before the development of IF/TA in kidney grafts. Such correlation between renal CB1 expression and renal fibrosis was never described in kidney grafts. We also suggested that CB1 is expressed by initially injured cells and by cells involved in synthesizing extracellular matrix proteins similarly to what is seen with the expression of DDR1, another important pathway in renal fibrosis.^47^ The high tubular expression of CB1 that we observed during CAD, using the exact same primary antibody and protocol that was previously used in the other types of CKD,^18^ enhances this hypothesis and strongly suggests a key role of CB1 in tubules in the IF/TA process.

To support this hypothesis, we tested whether CB1 expressed in tubules plays a causative role in the progression of fibrosis in an in vitro model mimicking CAD. Indeed, CAD is a multifactorial process in which a lot of immunological and non‐immunological causes are involved, including CNI toxicity. Since, it is impossible to completely reproduce the entire CAD process in vitro, we chose a simple model of epithelial‐to‐mesenchymal (EMT) transition in vitro. We therefore studied the effect of CNI on epithelial tubular cells because direct toxic effects of calcineurin inhibition on tubular function have been already well documented.^48^ Direct effects of CNI tubular toxicity include upregulation of TGFβ expression by tubular epithelial cells.^49^, ^50^, ^51^, ^52^ We developed a model of tacrolimus‐induced tubular injury and collagen synthesis in vitro which reproduces the first steps of CAD. We found increased expression of *cnr1*, *col3a1* and *col4a3* after treatment of mPETC by tacrolimus. Therefore, CB1 could be involved in the first steps of the development of CAD, possibly due to tacrolimus‐induced tubular epithelial injury. Interestingly, expression of *col3a1* and *col4a3* was significantly blunted by rimonabant, a CB1 antagonist (*P* < .05). Our results are in accordance with the recent literature where the specific deletion of CB1 in proximal tubules not only decreased renal fibrosis, injury and inflammation, but also preserved renal function in obesity‐induced nephropathy in mice.^22^

To conclude, our study strongly suggests an involvement of CB1 activation during CAD and paves the way to the development of CB1 inhibitors in CAD. However, the impact of cannabinoid system modulation on the evolution of chronic allograft dysfunction, as well as the cellular and molecular pathways involved, remains to be clarified.

## CONFLICT OF INTEREST

The authors confirm that there are no conflicts of interest.

## AUTHOR CONTRIBUTIONS

MD: conception of the work, data collection, data analysis and interpretation, drafting the article; LL: conception of the work, data collection, critical revision of the article; SV: data collection, data analysis and interpretation; MF: data collection, data analysis and interpretation; SF: data analysis and interpretation; KP: data collection; AD: revision of the article and Head of the Division of the Bicetre nephrology and transplantation department from which the patients were recruited; BH: data collection; CM: data collection; CC: conception of the work, critical revision of the article; HF: conception of the work, data collection, data analysis and interpretation, drafting the article. All authors reviewed the manuscript.

## Data Availability

‘The data that support the findings of this study are available from the corresponding author upon reasonable request.’
