# Supplementary figures and images for: The cannabinoid receptor 1 is involved in renal fibrosis during chronic allograft dysfunction: Proof of concept
Source: J Cell Mol Med. 2019 Aug 30;23(11):7279–88. doi: 10.1111/jcmm.14570 (PMC6815790; doi:10.1111/jcmm.14570)

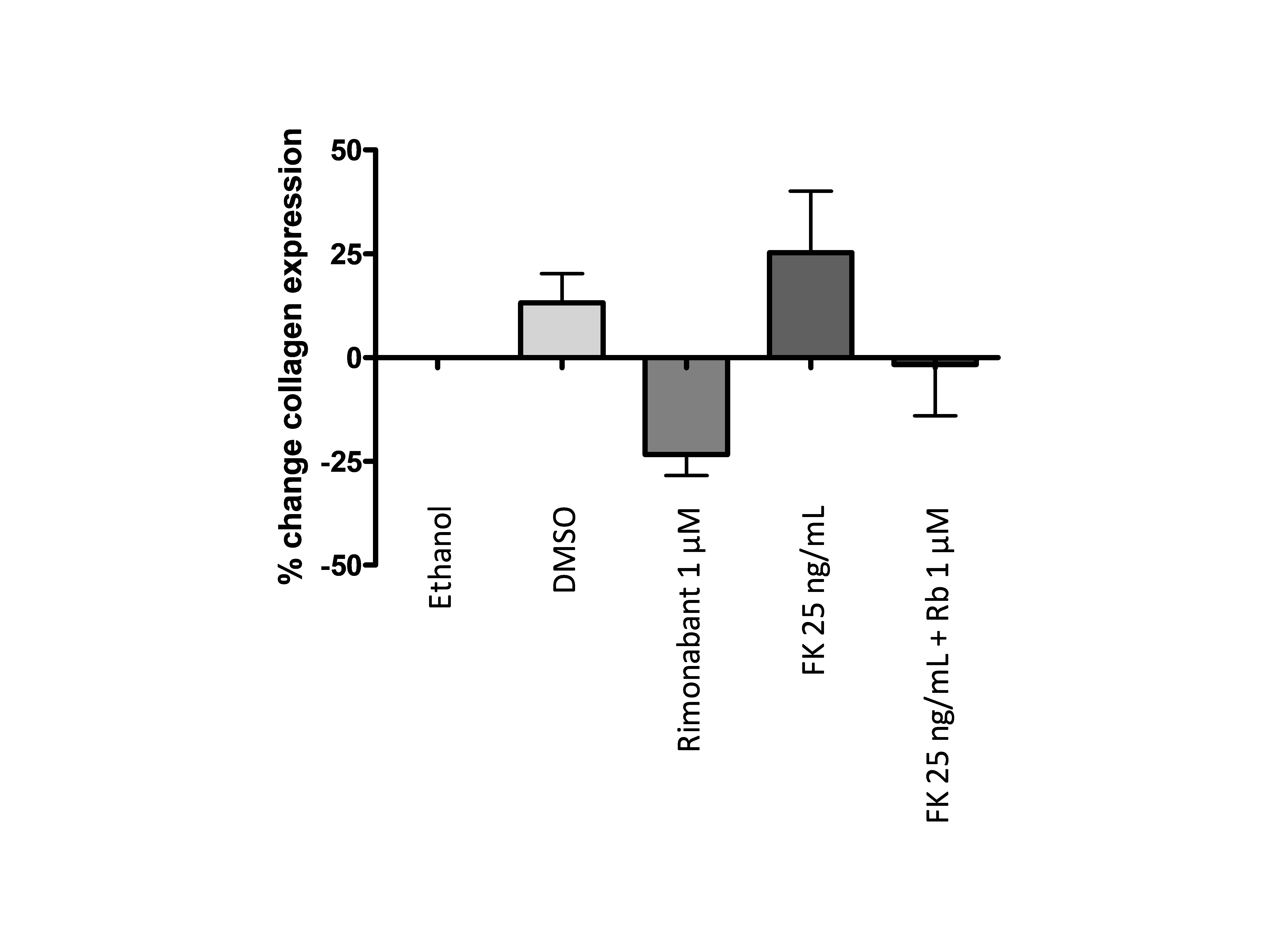

Supplement: Supplementary file 1 [file JCMM-23-7279-s001.tif]
