# Supplementary material for: The cannabinoid receptor 1 is involved in renal fibrosis during chronic allograft dysfunction: Proof of concept
Source: J Cell Mol Med. 2019 Aug 30;23(11):7279–88. doi: 10.1111/jcmm.14570 (PMC6815790; doi:10.1111/jcmm.14570)
Supplement: Supplementary file 2 [file JCMM-23-7279-s002.docx]

Supplementary table 1. Histological features of renal biopsies at D0 according to donor type

|  | CDD (n=4) | SD (n=8) | ECD (n=14) | *p* |
| --- | --- | --- | --- | --- |
| *Adequacy according to Banff recommendations* | | | | |
| Adequate samples (n) | 3 | 6 | 10 | 0.98 |
| Limit samples (n) | 1 | 2 | 3 | 0.98 |
| Inadequate samples (n) | 0 | 0 | 1 | 0.64 |
| *Acute lesions* | | | | |
| None of sample exhibited acute Banff lesions | | | | |
| Acute tubular necrosis | 100% | 100% | 71% | NS |
| *Chronic lesions* | | | | |
| Sclerotic glomeruli (%) | 0 ± 0 | 6.5 ± 5.7 | 15.5 ± 12.5 | 0.35 |
| Mesangial matrix increase « mm » | 0 ± 0 | 0 ± 0 | 0.21 ± 0.80 | 0.38 |
| Allograft glomerulopathy « cg » | 0 ± 0 | 0 ± 0 | 0 ± 0 | NA |
| Interstitial fibrosis « ci » | 0.25 ± 0.5 | 0.5 ± 0.53 | 0.85 ± 0.80 | 0.44 |
| Tubular atrophy « ct » | 0 ± 0 | 0.38 ± 0.52 | 0.54 ± 0.88 | 0.66 |
| Vascular fibrous intimal thickening « cv » | 0 ± 0 | 1 ± 0.93 | 1.14 ± 0.86 | 0.35 |
| Arteriolar hyaline thickening « ah » | 0.75 ± 0.50 | 0.29 ± 0.76 | 0.64 ± 1.1 | 0.60 |

Abbreviations: CCD, cardiac-dead donor; ECD, extended-criteria donor; SD, standard donor.

Digital data are means ± standard deviation
